# Meigs syndrome was misdiagnosed as a malignant ovarian tumor: a case report

**DOI:** 10.3389/fonc.2025.1624376

**Published:** 2025-09-05

**Authors:** Hongmei Li, Yanli Xing, Wei Hong, Yangyang Ma, Lizhi Niu

**Affiliations:** ^1^ Department of Oncology, Guangzhou Fuda Cancer Hospital, Guangzhou, China; ^2^ Department of Radiology, Guangzhou Fuda Cancer Hospital, Guangzhou, China; ^3^ Central Laboratory, Guangzhou Fuda Cancer Hospital, Guangzhou, China

**Keywords:** ovarian fibroma tumor, pleural effusion, ascites, CA125, Meigs syndrome

## Abstract

**Background:**

Meigs syndrome is characterized by the association of a benign ovarian tumor, typically an ovarian fibroma, with pleural effusion and ascites.

**Case summary:**

This report presents a case of a 54-year-old woman who was misdiagnosed with malignant ovarian neoplasm due to the presence of significant abdominal distension and elevated CA125 levels. Initial imaging at multiple external facilities suggested a left-sided malignant ovarian tumor, leading to unnecessary delays in treatment. Upon admission to our institution in April 2024, imaging confirmed a large pelvic mass, and subsequent diagnostic procedures indicated a likely fibroma. Surgical intervention revealed a left ovarian thecoma, and post-operative pathology confirmed the diagnosis. Notably, CA125 levels decreased from 335.1 U/ml to 164.6U/ml following surgery, and the patient showed significant clinical improvement.

**Conclusion:**

This case underscores the importance of considering Meigs syndrome in patients presenting with ovarian masses, pleural effusions, and elevated CA125, to prevent misdiagnosis and ensure timely management.

## Introduction

Meigs’ syndrome is a clinical condition characterized by the triad of an ovarian tumor, ascites, and pleural effusion, typically associated with benign ovarian tumors such as fibromas (1–3). This syndrome presents a diagnostic challenge, as the symptoms can closely mimic those of malignant ovarian tumors, leading to potential misdiagnosis and inappropriate treatment strategies. The incidence of Meigs’ syndrome is relatively low, with ovarian fibromas accounting for approximately 5-8% of all ovarian tumors, and only a small percentage of these cases presenting with the associated effusions (4). The elevation of cancer antigen 125 (CA125) levels is often observed in patients with ovarian tumors, further complicating the diagnostic landscape, as elevated CA125 can also indicate malignancy, thereby increasing the risk of misdiagnosis (5, 6).

The key differences between Meigs syndrome and ovarian malignancy include symptoms. Ovarian cancer may present with vaginal bleeding (either postmenopausal or intermenstrual), pelvic pain, early satiety, or weight loss, all of which are absent in our case. Moreover, malignant masses are typically fixed, irregular, and bilateral, whereas Meigs-associated mass are generally mobile, smooth, and unilateral. Furthermore, both conditions may show elevated CA125 levels, but in ovarian cancer, these levels usually rise progressively and correlate with tumor burden. In contrast, CA125 levels in Meigs syndrome return to normal after mass resection (7, 8).

In this case report, we present a rare instance of Meigs’ syndrome in a 54-year-old woman patient who was initially misdiagnosed with malignant ovarian cancer due to the presence of significant ascites and elevated CA125 levels. This case underscores the importance of considering Meigs’ syndrome as a differential diagnosis in patients presenting with abdominal masses, ascites, and elevated CA125, particularly when imaging studies suggest malignancy. Accurate recognition of this condition highlights the importance of intraoperative frozen section analysis. This analysis guides surgical radicality while preserving fertility or ovarian function when feasible, especially in young patients with suspected benign ovarian tumors.

## Case presentation

### Chief complaints

The patient is a 54-year-old woman who was hospitalized on April 29, 2024, after discovering a large abdominal and pelvic mass 18 months prior. She has a regular menstrual history, with menarche at 13 years old, a cycle length of 30 days, lasting 4–6 days, normal flow, and no dysmenorrhea or blood clots. She experienced menopause at age 52. Upon admission, physical examination revealed abdominal distension resembling that of an 8-month pregnancy, with negative shifting dullness and a healing incision from a peritoneal drainage tube. This case report was prepared in accordance with the CARE guidelines, and a completed CARE checklist is provided as **Supplementary File 1**.

### History of present illness

Nothing to declare.

### History of past illness

The patient had no history of hypertension, cardiovascular disease, type 2 diabetes mellitus, or coronary heart disease.

### Personal and family history

The patient denies a history of familial genetic diseases, tumors, infectious diseases, and psychiatric disorders.

### Physical examination

Nothing to declare.

### Laboratory examinations

Serum CA125 levels were measured at 335.1 U/ml.

### Imaging examinations

**Figure 1:**
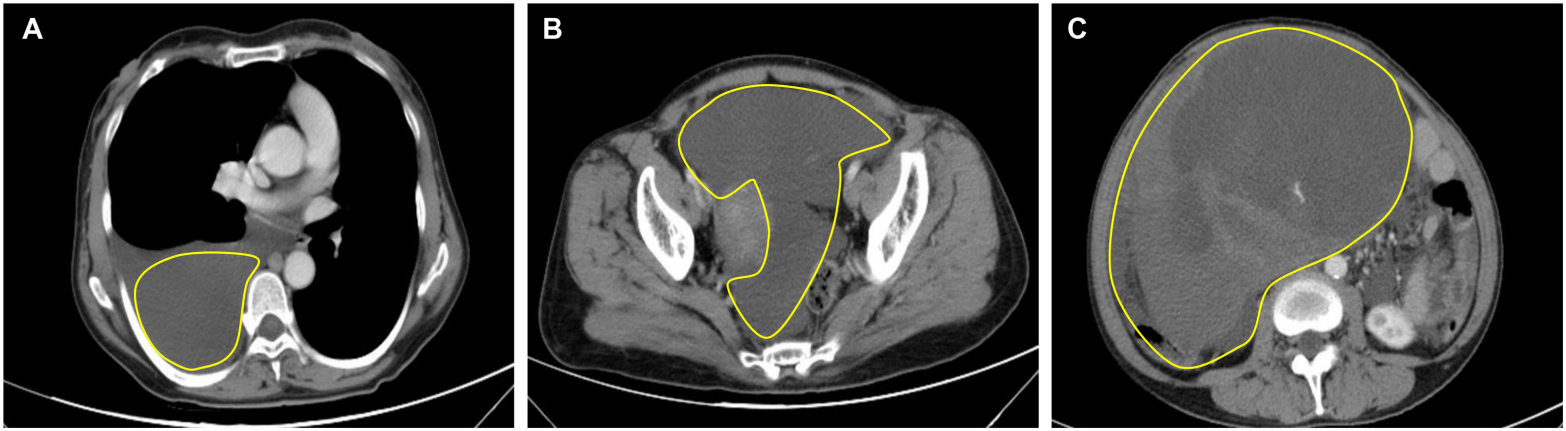
Preoperative CT of the thorax and abdomen. **(A)** CT thorax showed right-sided pleural effusion; **(B)** Abdomen CT showed ascites; **(C)** Transverse view of the abdomen shows a large solid tumor measuring 21.5 × 14.6 cm.

Computed tomography (CT) of the chest showed significant pleural effusion and ascites, (**Figures 1A, B**). Transverse view of the abdomen showed a large solid mass measuring 21.5 × 14.6 cm (**Figure 1C**).

### Final diagnosis

Meigs’ syndrome.

### Treatment

**Figure 2:**
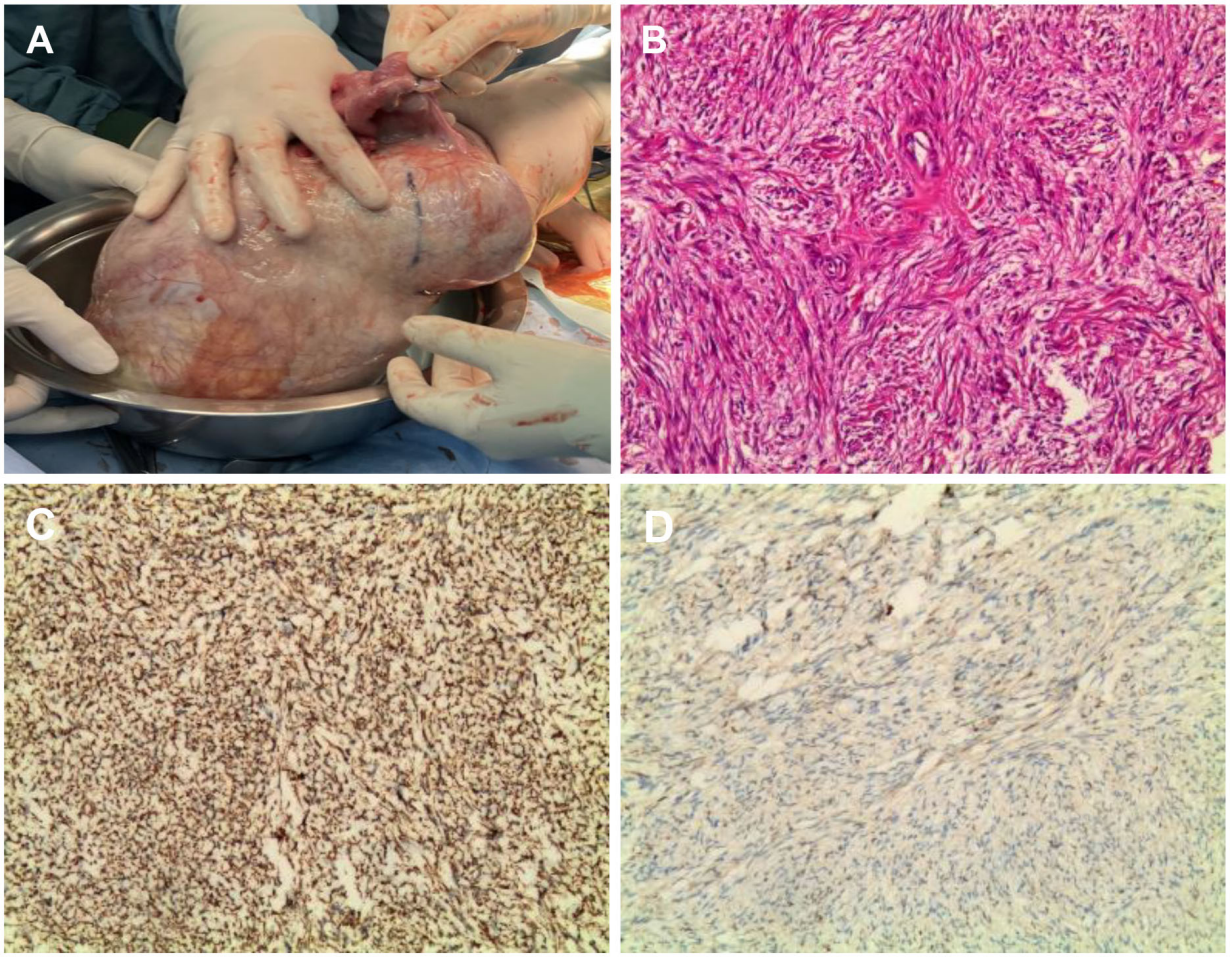
Intraoperative images of the tumor. **(A)** Intraoperative view of the tumor during open laparotomy; **(B)** Hematoxylin and eosin (H&E) stained histological; **(C)** Immunohistochemistry showed Inhibin-a(+); **(D)** Immunohistochemistry showed Vim(3+).

**Figure 3:**
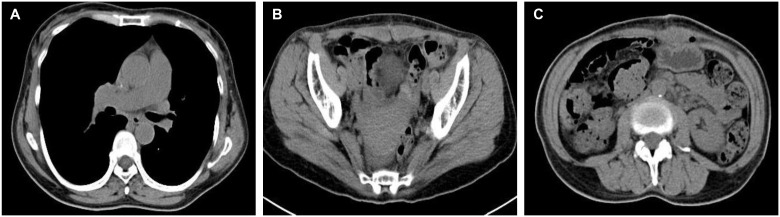
Post 1 week CT of the thorax and abdomen. **(A)** CT chest showed complete resolution of right-sided pleural effusion; **(B)** Abdominal CT showed the disappearance of ascites; **(C)** Abdominal CT showed no sign of tumor recurrence.

On April 30, 2024, the patient underwent surgical treatment, including skin incision, electrosurgical incision of subcutaneous fascia, and blunt dissection of the rectus abdominis muscle. The peritoneum was opened, revealing approximately 200 ml of clear, pale yellow ascitic fluid. The uterus appeared normal, and the right ovary and fallopian tube were unremarkable. The left ovary was significantly enlarged, presenting a mass measuring 25 × 20 × 20 cm, with an intact capsule and predominantly solid characteristics. The left fallopian tube was thickened and adhered to the mass, prompting a left adnexectomy (**Figures 2A**). Hemostasis was achieved by clamping the pelvic funnel ligament, isthmus of the fallopian tube, and ovarian ligament, followed by cutting and suturing. The left adnexa was sent for frozen section examination, which suggested benign characteristics. A thorough exploration of the abdominal cavity revealed no other abnormalities. Post-operative pathology demonstrated: immunohistochemistry results of CD10 (+), CD117 (-), CEA (+), Desmin (-), DOG-1 (-), EGFR (+), ER (-), Ki-67 (approximately 1%+), P53 (-), PAX-8 (-), PR (+), S-100 (-), SMA (-), Vim (+), VEGF (-), and inhibin-a (+). The pathological results confirmed it as a follicular membrane tumor of the left ovary, with no sign of malignant tumor (**Figures 2B–D**). The final diagnosis was Meigs syndrome. The patient was discharged on May 11, 2024, with good post-operative recovery. Nine days post-surgery, the patient’s CA125 level decreased to 164.6 U/ml. Post 1 week CT of the thorax and abdomen showed complete resolution of pleural effusion and ascites (**Figures 3A, B**), abdominal CT showed no sign of tumor recurrence (**Figures 3C**). Two weeks post-surgery, the CA125 level returned to normal.

### Outcome and follow-up

She remained disease-free at a 1-year follow-up.

## Discussion

The case presented highlights a significant diagnostic challenge associated with Meigs’ syndrome, particularly its potential to be misdiagnosed as malignant ovarian tumors. Meigs’ syndrome is characterized by the triad of an ovarian tumor, ascites, and pleural effusion, often linked with benign tumors such as ovarian fibromas. This misdiagnosis can have profound implications for clinical management and patient outcomes, as surgical interventions intended for malignancy may not be necessary when the underlying condition is benign (9).

In reviewing the literature, it becomes evident that ovarian fibromas, while constituting only 5-8% of all ovarian tumors, can present in ways that mimic malignant conditions. Approximately 10-15% of these fibromas are associated with ascites or pleural effusion, leading to the clinical picture of Meigs’ syndrome (10). Cha et al. conducted an analysis of 43 cases derived from 33 publications spanning the years 1989 to 2012. Their findings revealed 22 instances of fibroma (which included cellular fibroma), 7 occurrences of fibrothecoma, 4 cases of thecoma (comprising luteinized thecoma), 3 examples of granulosa cell tumor (including its juvenile variant), 2 instances of Brenner tumor, and 5 occurrences of sclerosing stromal tumor, the latter of which did not fulfill the conventional criteria for Meigs’ syndrome (11). In this patient’s case, multiple imaging modalities, including CT and MRI, indicated a high likelihood of malignancy, which ultimately proved incorrect upon pathological examination. Previous studies have similarly demonstrated that imaging findings such as large solid masses and ascites can lead to a presumption of malignancy, necessitating careful consideration of benign differentials (4). The critical features differentiating Meigs syndrome from ovarian carcinoma are summarized in **Table 1**.

**Table 1:** Differential features between Meigs syndrome and ovarian cancer.

Category	Meigs syndrome	Ovarian cancer
Clinical Features		
Age	Perimenopausal (50-60 years)	Wide range (peak 60-65 years)
Key triad	Ovarian mass + pleural effusion + ascites	Pelvic mass + ascites ± distant metastases
Vaginal bleeding	Rare	Common
CA125	Elevated but normalizes post-surgery	Persistently elevated or progressive rise
Diagnostic Features		
Tumor characteristics	Unilateral, Smooth margins	Often bilateral, Irregular/necrotic
Imaging findings	No peritoneal implants, No lymphadenopathy	Peritoneal carcinomatosis, LN metastasis
Cytology of effusion	Negative for malignancy	Positive for malignancy
Pathology		
Tumor type	Benign fibroma/thecoma	Malignant epithelial tumor
Immunohistochemistry	Inhibin-α^+^, ER/PR^+^	WT1^+^ (serous), p53^+^
Treatment		
Primary management	Surgical resection of tumor	Debulking surgery + chemotherapy
Adjuvant therapy	Not required	Platinum-based regimens
Prognosis		
Risk of recurrence	<5%	70% (advanced stage)
5-year survival	100%	40-50% (stage III-IV)

The patient’s elevated CA125 levels further complicated the diagnostic process. CA125 is widely recognized as a tumor marker for ovarian cancer, but it can also be elevated in benign conditions, including Meigs’ syndrome, endometriosis, and pelvic inflammatory disease (12–14). The case underscores the necessity for clinicians to interpret elevated CA125 levels within a broader clinical context and to consider benign etiologies when presented with associated findings, such as significant ascites and effusion. The mechanism of CA-125 elevation in Meigs’ syndrome is unclear. Liou et al. suggest it’s due to mesothelial rather than tumor expression, as tumor specimens were CA-125 negative (15). Studies on CA-125 levels and disease presentation show mixed results: Shang found a positive correlation with ascites volume, while Iavarone and Kortekaas found no correlation, though the latter noted a potential link with hydrothorax (16–18). Despite our patient having high CA-125 levels with massive ascites and pleural effusion, no correlation was found in the literature (19, 20).

The patient’s elevated CA125 levels further complicated the diagnostic process. CA-125 is a glycoprotein produced by mesothelial cells and serves as a tumor marker for ovarian cancer, but its elevation is nonspecific and can occur in various benign and malignant conditions. Elevated CA-125 levels can be found in benign situations. These include peritoneal irritation, inflammatory conditions, and benign ovarian tumors like fibromas and thecomas (17, 21, 22). Notably, pleural effusion and ascites themselves can stimulate mesothelial cells to release CA125, independent of malignancy (23). Malignant causes extend beyond ovarian cancer to include gastrointestinal, endometrial, and even mesothelioma. This highlights that CA125 must be interpreted in conjunction with imaging, histopathology, and symptom constellation.

This case emphasizes the critical need for thorough diagnostic evaluation, including histopathological assessment, to avoid misdiagnosis. The distinction between benign and malignant ovarian masses can be challenging, particularly when imaging results are suggestive of malignancy. Pathological examination remains the gold standard for definitive diagnosis, as imaging alone may not provide sufficient clarity (24). The case illustrates the importance of considering Meigs’ syndrome in patients with ovarian masses and associated symptoms, particularly when faced with elevated CA125 levels and significant fluid collections.

The strength of this case lies in its comprehensive documentation of a diagnostically challenging case that integrates multimodal clinical data, including serial CA125 monitoring, radiological-pathological correlation, and long-term follow-up. We provide empirical evidence supporting the benign nature of Meigs syndrome despite malignancy-mimicking features. However, several limitations should be acknowledged. Firstly, the single-case design inherently restricts generalizability. Secondly, preoperative PET imaging which could have provided additional metabolic characterization was not performed, a limitation noted in similar studies where PET-CT helped differentiate benign from malignant lesions. Thirdly, while CA125 dynamics were meticulously tracked, other potential biomarkers like HE4 (Human Epididymis Protein 4) were not assessed, despite emerging evidence of their complementary role in distinguishing benign from malignant effusions. Future multi-center studies with larger cohorts are warranted to validate these findings and establish standardized diagnostic algorithms for Meigs syndrome.

In conclusion, the lessons learned from this case should inform clinical practice moving forward. Clinicians must maintain a high index of suspicion for Meigs’ syndrome in similar presentations and approach elevated CA125 levels with caution, recognizing the potential for benign conditions. Future research should aim to enhance diagnostic methodologies for ovarian tumors, particularly in distinguishing between benign and malignant pathologies, thereby improving patient care and outcomes (25).

## Conclusion

This case highlights the diagnostic challenge of distinguishing Meigs syndrome from malignancy in patients with an ovarian mass, massive ascites, and markedly elevated CA125 (>2000 U/mL), emphasizing the pivotal role of intraoperative frozen section in guiding conservative (fertility-sparing) versus radical surgery. Our findings support a two-stage surgical approach—initial tumor resection with frozen biopsy, followed by definitive staging if malignancy is confirmed—to balance diagnostic certainty and overtreatment risks, particularly in young patients. Further multicenter studies are needed to validate frozen section accuracy in giant (>20 cm) ovarian tumors and refine evidence-based surgical algorithms.

## Data Availability

The raw data supporting the conclusions of this article will be made available by the authors, without undue reservation.

## References

[1] Fernandez Diaz JJ Navarro Desentre L . Meigs’ Syndrome. N Engl J Med. (2024) 390:2107. doi: 10.1056/NEJMicm2313447, PMID: 38856181

[2] Tan Q Xu LF Yan T Huang CH Tao Y Huang WH . Deciphering the puzzle: a case report of Tjalma syndrome (pseudo-pseudo Meigs’ syndrome) with profoundly elevated CA-125 and pleural effusion. Front Immunol. (2023) 14:1277683. doi: 10.3389/fimmu.2023.1277683, PMID: 38162662 PMC10756667

[3] Meigs JV . Fibroma of the ovary with ascites and hydrothorax; Meigs’ syndrome. Am J Obstet Gynecol. (1954) 67:962–85. doi: 10.1016/0002-9378(54)90258-6, PMID: 13148256

[4] Dalal N Athwal PSS Tharu B Saravanan L Mansour H . A rare case of pseudo-meigs’ Syndrome with ovarian metastasis presenting as meigs’ Syndrome. Cureus. (2020) 12:e11022. doi: 10.7759/cureus.11022, PMID: 33214950 PMC7671297

[5] Liu J Li L Li Y Wang Q Liu R Ding H . Regional metabolic and network changes in Meige syndrome. Sci Rep. (2021) 11:15753. doi: 10.1038/s41598-021-95333-8, PMID: 34344985 PMC8333318

[6] Zou L Lou J Huang H Xu L . Pseudo-Meigs syndrome caused by a rapidly enlarging hydropic leiomyoma with elevated CA125 levels mimicking ovarian Malignancy: a case report and literature review. BMC Womens Health. (2024) 24:445. doi: 10.1186/s12905-024-03285-8, PMID: 39112955 PMC11304927

[7] Navarro-Esteva J Laseca-Modrago M Arencibia-Sánchez O . Two patients with meigs’ Syndrome and elevated serum CA-125: A case report. Cureus. (2020) 12:e8927. doi: 10.7759/cureus.8927, PMID: 32760627 PMC7392357

[8] Li Q Tian B . Case report: Three cases of systemic lupus erythematosus presenting primarily with massive ascites and significantly elevated CA-125 levels and a review of pseudo-pseudo Meigs’ syndrome in literature. Front Immunol. (2024) 15:1423631. doi: 10.3389/fimmu.2024.1423631, PMID: 39081322 PMC11286392

[9] Abdelgawad M Barghuthi L Davis T Omar M Kamel OM Gibbons J . Large uterine leiomyoma presenting as pseudo-Meigs’ syndrome with an elevated ca125: a case report and literature review. J Surg Case Rep. (2022) 2022:rjac253. doi: 10.1093/jscr/rjac253, PMID: 35685293 PMC9173737

[10] Roecker ZA Young MR Han C . Rapidly progressing ascites in a pregnancy with a massive fibroid: A case report and review of pseudo-Meigs syndrome. Int J Gynecol Obstet. (2024) 167:128–31. doi: 10.1002/ijgo.15604, PMID: 38736303

[11] Cha MY Roh HJ You SK Lee SH Cho HJ Kwon YS . Meigs’ syndrome with elevated serum CA 125 level in a case of ovarian fibrothecoma. Eur J Gynecol Oncol. (2014) 35:734–7. PMID: 25556284

[12] Gruson D Maisin D Pouleur A-C Ann SA Rousseau MF . CA125, Galectin-3 and FGF-23 are interrelated in heart failure with reduced ejection fraction. EJIFCC. (2023) 34:103–9. PMID: 37455845 PMC10349309

[13] Morán-Mendoza A Alvarado-Luna G Calderillo-Ruiz G Serrano-Olvera A López-Graniel CM Gallardo-Rincón D . Elevated CA125 level associated with Meigs’ syndrome: case report and review of the literature. Int J Gynecol Cancer. (2006) 16 Suppl 1:315–8. doi: 10.1111/j.1525-1438.2006.00228.x, PMID: 16515612

[14] Yuan L Cui L Wang J Gong L . A case report of meigs’ Syndrome caused by ovarian fibrothecoma with high levels of CA125. Int J Womens Health. (2024) 16:519–25. doi: 10.2147/IJWH.S450833, PMID: 38544782 PMC10967536

[15] Liou J-H Su TC Hsu J-C . Meigs’ syndrome with elevated serum cancer antigen 125 levels in a case of ovarian sclerosing stromal tumor. Taiwan J Obstet Gynecol. (2011) 50:196–200. doi: 10.1016/j.tjog.2011.01.011, PMID: 21791307

[16] Shang W Wu L Xu R Chen X Yao S Huang P . Clinical laboratory features of Meigs’ syndrome: a retrospective study from 2009 to 2018. Front Med. (2021) 15:116–24. doi: 10.1007/s11684-019-0732-6, PMID: 32651935

[17] Iavarone I Padovano M Pasanisi F Della Corte L La Mantia E Ronsini C . Meigs syndrome and elevated CA-125: case report and literature review of an unusual presentation mimicking ovarian cancer. Medicina (Kaunas). (2023) 59:1684. doi: 10.3390/medicina59091684, PMID: 37763803 PMC10535830

[18] Kortekaas KE Pelikan HM . Hydrothorax, ascites and an abdominal mass: not always signs of a Malignancy - Three cases of Meigs’ syndrome. J Radiol Case Rep. (2018) 12:17–26. doi: 10.3941/jrcr.v12i1.3209, PMID: 29875983 PMC5965394

[19] Potiris A Pateras IS Koufopoulos N Samaras MG Topis S Chousmekeridou M-G . Rare giant ovarian thecoma presented as atypical/incomplete Meigs’ syndrome: A case image report. Clin Case Rep. (2024) 12:e9558. doi: 10.1002/ccr3.9558, PMID: 39493794 PMC11527809

[20] Dellaportas D Kollia D Myoteri D Nastos C Gkiokas G Carvounis E . Giant ovarian thecoma associated with meigs syndrome: A striking case. Chirurgia (Bucur). (2021) 116:1–5. doi: 10.21614/chirurgia.116.eC.1912, PMID: 34463241

[21] Zager Y Khalilieh S Mansour A Cohen K Nadler R Anteby R . The value of CA125 in predicting acute complicated colonic diverticulitis. Int J Colorectal Dis. (2023) 38:182. doi: 10.1007/s00384-023-04478-7, PMID: 37389666

[22] Wang X Albayda J Paik JJ Tiniakou E Adler B Mammen AL . Evaluating CA-125 and PET/CT for cancer detection in idiopathic inflammatory myopathies. Rheumatol (Oxford). (2025) 64:2115–22. doi: 10.1093/rheumatology/keae470, PMID: 39222439 PMC11962979

[23] He J Li J Fan B Yan L Ouyang L . Pseudo-pseudo Meigs syndrome in systemic lupus erythematosus misdiagnosed as pseudo-Meigs’ syndrome: A case report. J Obstet Gynecol Res. (2023) 49:2199–204. doi: 10.1111/jog.15677, PMID: 37199068

[24] Liang J He X Lu J . Multiple system atrophy associated with Meige syndrome: A rare case report. Radiol Case Rep. (2023) 18:3192–5. doi: 10.1016/j.radcr.2023.04.055, PMID: 37448600 PMC10338191

[25] Akasbi I Akammar A Ezzoulali Z Ouazzani Chahdi H Chaouche I Iraqui Houssaini E . Demons-Meigs syndrome: Rare cause of intraperitoneal and pleural effusion. Radiol Case Rep. (2025) 20:3067–3071. doi: 10.1016/j.radcr.2025.03.033, PMID: 40242384 PMC12002815

