# Supplementary material for: Meigs syndrome was misdiagnosed as a malignant ovarian tumor: a case report
Source: Front Oncol. 2025 Sep 5;15:1624376. doi: 10.3389/fonc.2025.1624376 (PMC12446027; doi:10.3389/fonc.2025.1624376)
Supplement: Supplementary file 1 [file DataSheet1.pdf]

CARE Checklist – 2016: Information for writing a case report

Topic	Item	Checklist item description	Line/Page
**Title**	1	The words "case report" should be in the title along with the area of focus	1/1
**Key Words**	2	Four to seven key words—include "case report" as one of the key words	32/2
**Abstract**	3a	Background: What does this case report add to the medical literature?	17/2
	3b	Case summary: chief complaint, diagnoses, interventions, and outcomes	20/2
	3c	Conclusion: What is the main "take-away" lesson from this case?	28/2
**Introduction**	4	The current standard of care and contributions of this case—with references (1-2 paragraphs)	48/3
**Timeline**	5	Information from this case report organized into a timeline (table or figure)	77/4
**Patient Information**	6a	De-identified demographic and other patient or client specific information	76/3
	6b	Chief complaint—what prompted this visit?	76/3
	6c	Relevant history including past interventions and outcomes	86/4
**Physical Exam**	7	Relevant physical examination findings	97/4
**Diagnostic Assessment**	8a	Evaluations such as surveys, laboratory testing, imaging, etc.	108/5
	8b	Diagnostic reasoning including other diagnoses considered and challenges	108/5
	8c	Consider tables or figures linking assessment, diagnoses and interventions	108/5
	8d	Prognostic characteristics where applicable	108/5
**Interventions**	9a	Types such as life-style recommendations, treatments, medications, surgery	111/5
	9b	Intervention administration such as dosage, frequency and duration	118/5
	9c	Note changes in intervention with explanation	124/5
	9d	Other concurrent interventions	128/5
**Follow-up and Outcomes**	10a	Clinician assessment (and patient or client assessed outcomes when appropriate)	132/5
	10b	Important follow-up diagnostic evaluations	132/5
	10c	Assessment of intervention adherence and tolerability, including adverse events	132/5
**Discussion**	11a	Strengths and limitations in your approach to this case	189/7
	11b	Specify how this case report informs practice or Clinical Practice Guidelines (CPG)	144/6
	11c	How does this case report suggest a testable hypothesis?	127/5
	11d	Conclusions and rationale	203/8
**Patient Perspective**	12	When appropriate include the assessment of the patient or client on this episode of care	139/5
**Informed Consent**	13	Informed consent from the person who is the subject of this case report is required by most journals	224/8
**Additional Information**	14	Acknowledgement section; Competing Interests; IRB approval when required	240/9
